# Supplementary material for: Age-related differences in trust beliefs during middle childhood: Downward-extension and validation of the general trust scale
Source: PLoS One. 2025 May 30;20(5):e0322790. doi: 10.1371/journal.pone.0322790 (PMC12124571; doi:10.1371/journal.pone.0322790)
Supplement: S1 Appendix — (DOCX) [file pone.0322790.s001.docx]

Appendix: Trust Definition Codebook

Examples of the types of responses that would and would not be coded as describing the construct of “trust”, and example responses for each of the components of uncertainty/risk, reliability, honesty, and emotional trust, respectively.

**Trust Definitions**

Potential responses that define *trust*:

- Knowing you can depend on that person to help you
- When you believe other people will tell the truth to you
- Relying on people to do what they said they would
- Believing others will do as promised
- To rely on someone
- You trust people if you ask them to do something for you and you know they will get it done
- If you tell someone a secret and you know they won’t share it with anyone, you trust them
- If you know someone would help you just because you asked them to, you know you can trust that person

Potential responses that do *not* meet the definition:

- Believing in someone, or believing someone
- Being honest [in the absence of a description of *believing* people will be, or generally are, honest]
- Understanding a person
- Knowing that a person cares for/about you
- When you’re good friends with them
- Putting hope in someone else
- If you think they will do it, you trust them
- It means you can trust someone, like a close friend
- Being honest so people trust you
- When people are nice to you or buy you gifts

**Uncertainty/Risk**

Potential responses that describe the component:

- If you gave someone your favourite thing even though if it broke you would cry, you know you can trust them
- They will always be honest even if it gets them in trouble
- You trust someone if you tell them something you don’t want anyone else to know

**Reliability**

Potential responses that describe the component:

- Having faith in someone/faith in other people
- Believing you can depend on someone
- When you know you can rely on someone
- People you trust will always be there for you
- If you ask that person to do something for you, you know they will do it

**Honesty**

Potential responses that describe the component:

- When you know people won’t lie to you
- Someone will tell the truth if you ask them a question
- You can trust people who are honest with you

**Emotional Trust**

Potential responses that describe the component:

- When you know it’s safe to tell them a secret
- Knowing if you tell people something and ask them not to share, they won’t tell anyone else
- They won’t try to make you feel bad
- Being sure people won’t break their promises to you
- You can rely on someone to do things like keep your secrets
- You trust someone if you can you tell them something and you know that they won't go away and tell someone else
